# Dehydration of bacterial cellulose and the water content effects on its viscoelastic and electrochemical properties

**DOI:** 10.1080/14686996.2018.1430981

**Published:** 2018-03-09

**Authors:** Ana R. Rebelo, Andrew J. Archer, Xiuli Chen, Changqing Liu, Guang Yang, Yang Liu

**Affiliations:** a Centre of Biological Engineering, Wolfson School, Loughborough University, Loughorough, UK; b Wolfson School of Mechanical, Electrical and Manufacturing Engineering, Loughborough University, Loughborough, UK; c Department of Mathematical Sciences, Loughborough University, Loughborough, UK; d Department of Biomedical Engineering, College of Life Science and Technology, Huazhong University of Science and Technology, Wuhan, P.R. China

**Keywords:** Bacterial cellulose, dehydration, mechanical, electrochemical, 20 Organic and soft materials (colloids, liquid crystals, gel, polymers), 102 Porous / Nanoporous / Nanostructured materials, 211 Scaffold / Tissue engineering / Drug delivery

## Abstract

Bacterial cellulose (BC) has interesting properties including high crystallinity, tensile strength, degree of polymerisation, water holding capacity (98%) and an overall attractive 3D nanofibrillar structure. The mechanical and electrochemical properties can be tailored upon incomplete BC dehydration. Under different water contents (100, 80 and 50%), the rheology and electrochemistry of BC were evaluated, showing a progressive stiffening and increasing resistance with lower capacitance after partial dehydration. BC water loss was mathematically modelled for predicting its water content and for understanding the structural changes of post-dried BC. The dehydration of the samples was determined via water evaporation at 37 °C for different diameters and thicknesses. The gradual water evaporation observed was well-described by the model proposed (*R* ^2^ up to 0.99). The mathematical model for BC water loss may allow the optimisation of these properties for an intended application and may be extendable for other conditions and purposes.

## Graphical abstract

**Figure:**
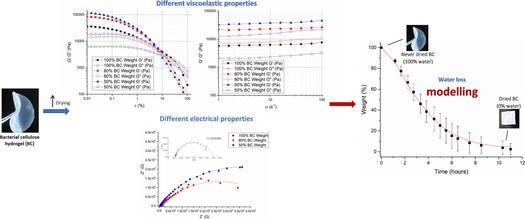

## 1. Introduction

Bacterial cellulose (BC) is a type of cellulose with a 3D nanofibrillar structure commonly produced by fermentation of *Acetobacter xylinum* bacteria in static or agitated cultures [1,2]. In contrast to plant cellulose, BC does not require prior purification steps ascribed to the absence of impurities and contaminants, which renders BC a high polymerisation degree ready for industrial application at low cost [2]. Due to abundant hydrogen bonds, BC retains large amounts of water (around 99%), hence, presented as a hydrogel. Additionally, the hydroxyl groups also interact with each other, establishing intra- and inter-molecular hydrogen bonds to form amorphous and crystalline domains, respectively [3,4]. Because of the higher number of crystalline domains, BC is very strong under tensile load yet flexible and insoluble in almost all solvents, including water [4]. The numerous hydroxyl groups contribute further to the reactivity of BC, allowing straightforward modification and functionalisation via incorporation of monomeric and/or other reactive species, such as of polymers, carbon nanotubes, graphene and metal nanoparticles, into the BC network [5]. Another advantage of BC lies in its good biocompatibility and the nano-scale network of fibril structure that mimics to some extent the structure of extracellular matrix of mammalian tissues. These attribute to its biocompatibility and, hence, it had been approved by the US Food and Drug Administration (FDA) for biomedical applications [6], such as wound dressings, tissue engineering, biocapacitors, bio-batteries, and also in food, electronics and paper industries [7,8].

Although extensively studied in the last few years, the properties of BC are far from being completely understood, in particular how the content of bounded and unbounded water would be reduced during the drying process and its corresponding effects on its viscoelastic and electrochemical properties. This paper intends to elucidate some of these aspects of BC and the influence of the BC membrane’s geometry. In previous works, a mathematical model was designed for predicting the time-dependent rheological behaviour of never-dried BC membranes [9] and that of nanocellulose suspensions [10]. Hygroscopic power of amorphous cellulose was also studied upon swelling to show the effects of hydration on a number of physical parameters of amorphous cellulose [11]. A theoretical modelling was proposed for water vapour transport in cellulose-based materials for applications in food and paper packaging [12]. However, to the best of our knowledge, none has studied the evaporation process of BC membranes, in particular considering the influence of water content effects on its viscoelastic and electrochemical properties. Herein, a mathematical model is also proposed for analysis and eventual predication of the water loss of BC membranes at 37 °C, with a determined thickness and surface area. This analysis will be important in terms of maximising or optimising the properties interested for a specific application.

## 2. Material and methods

### 2.1. BC synthesis

BC membranes were produced from the fermentation of Gluconacetobacter hansenii (ATCC® 53582TM, Manassas, VA, USA). The bacteria strain was inoculated at 10% in steam sterilised (at 121 °C for 20 min) culture medium containing disodium hydrogen phosphate dodecahydrate (6.8 g/L), peptone (5 g/L), yeast extract (5 g/L), citric acid (1.5 g/L), glucose (20 g/L) and pure water. Gluconacetobacter hansenii was cultured in sterile environment in 6- and 24-well plates, maintaining static cultivation for 4 days and 2 weeks to get different thicknesses. The thicknesses of the obtained BC pellicles measured between 2 and 8 mm, respectively. Purification was done by washing the membranes with pure water for 2–3 days, prior to boiling with sodium hydroxide solution (4 g/L) for 40 min to remove medium and any adsorbed bacteria. The morphological and chemical characteristics (see Figure S1 in supplemented data) and fibre sizes of BC used in this study were similar to those reported in literature [9,13–15].

### 2.2. Viscoelastic properties

The viscoelastic properties of BC membranes with 100, 80 and 50% of initial water content, which corresponds to 98, 78.4 and 49% of BC total weight respectively, were assessed with a parallel plate rheometer (Physica MCR, Anton Paar, St Albans, UK). Three BC membrane samples per each water content levels were cut into squares of 2.5 × 2.5 cm^2^ and placed on the plate at 37 °C, to simulate the human body temperature. Based on the weight-loss curves, the samples with 80 and 50% water content were obtained by placing fully swollen BC membranes in the incubator at 37 °C for the different time periods required to allow loss of 20 and 50% of their initial weight, respectively. Never-dried fully swollen membranes measured 2.3 ± 0.4 mm of thickness. The tests were performed in amplitude and frequency sweep modes and storage (*G*′) and loss (*G*″) moduli as function of strain (*ε*) and angular frequency (*ω*) were obtained.

### 2.3. Electrochemical properties

Electrochemical properties of BC membranes were assessed through electrochemical impedance spectroscopy. The spectra of never-dried (100% water content) and partially dried (80 and 50% water content) BC membranes were acquired using Galvanostat/Potentiostat (Eco Chemie microAutolab type III, KM Utrecht, The Netherlands). The data were recorded for 0 V of DC potential and on superimposition of a sinusoidal AC potential of 10 mV, over the frequency range of 9997 Hz–0.1 Hz. The samples used were roughly 8.0 ± 0.5 mm thick in their never-dried state and 8 mm in diameter (obtained using a standard biopsy punch).

### 2.4. Dehydration and modelling

Five ready-made BC membranes per thickness (8.0 ± 0.5 mm and 2.0 ± 0.2 mm) and per size (8 and 5 mm diameter obtained using standard biopsy punches) were kept in an incubator (Sanyo MCO-18AIC CO2, Boston, MA, USA) at 37 °C and 5% CO_2_). At different time points, the samples were weighted using a high precision scale electronic balance (Adventurer® Pro, Ohaus, Parsippany, NJ, USA). Before weighing, the excess water on the surface of the ready-made samples was carefully removed by tapping onto paper towels. The term TxDy is used to designate the geometry of samples, i.e. membranes with thickness Tx (*x* = 2 or 8 mm) and diameter Dy (*y* = 5 or 8 mm). Herein, the terms ‘weight loss’ and ‘water loss’ are used interchangeably to refer to dehydration process of BC samples, as BC weight-loss at 37 °C is only due to water evaporation.

In order to model the BC weight-loss via water evaporation, a simple model is proposed based on the hypothesis that the water in the system assumes one of the three following states: bound (*B*), free (*F*) or evaporated (*E*). Rate equations were used to describe the transitions between the three different states, as shown in Equation (1), where *k*_1_ is the rate coefficient for bound water becoming free, *k*_2_ the rate coefficient for free water becoming bound and *k*_3_ the rate coefficient for free water evaporating.

(1) $$B\overset{k_{1}}{\underset{k_{2}}{⇄}}F\overset{k_{3}}{\underset{}{\to }}E$$

Considering that, from experimental observation, the process involved in the conversion of bound water to free water is reversible and that the conversion of free water to evaporated is irreversible (i.e. we assume there is no condensation of water from the atmosphere into the BC), differential equations may be written for calculating the amount of bound water and free water as a function of time (*B*(*t*) and *F*(*t*), respectively), following the thermodynamic laws.

(2) $$\begin{array}{cc} \frac{\text{d}}{\text{d}t}B\left(t\right) & =k_{2}F\left(t\right)-k_{1}B\left(t\right)\\ \text{and} & \\ \frac{\text{d}}{\text{d}t}F\left(t\right) & =-k_{1}B\left(t\right)+k_{2}F\left(t\right)-k_{3}F\left(t\right) \end{array}$$

It is then straightforward to solve these coupled linear differential equations; herein the Maple computer algebra package is used. The total mass of the system equals the sum of the individual masses of both bound and free water, along with that of the net mass of cellulose membrane. Considering that only 2% of the total mass of BC is that of the cellulose, the total mass fraction is given by the sum:

(3) M=B+F+0.02.

OriginLab software was used to determine the parameters *k*_1_, *k*_2_ and *k*_3_ recurring to the damped least-squares. The difference between the observed and estimated value (Residuals or R) and the respective coefficient of determination (R-squared or *R* ^2^) of the best fitting obtained were calculated and discussed accordingly.

## 3. Results

### 3.1. Viscoelastic properties

**Figure 1.**
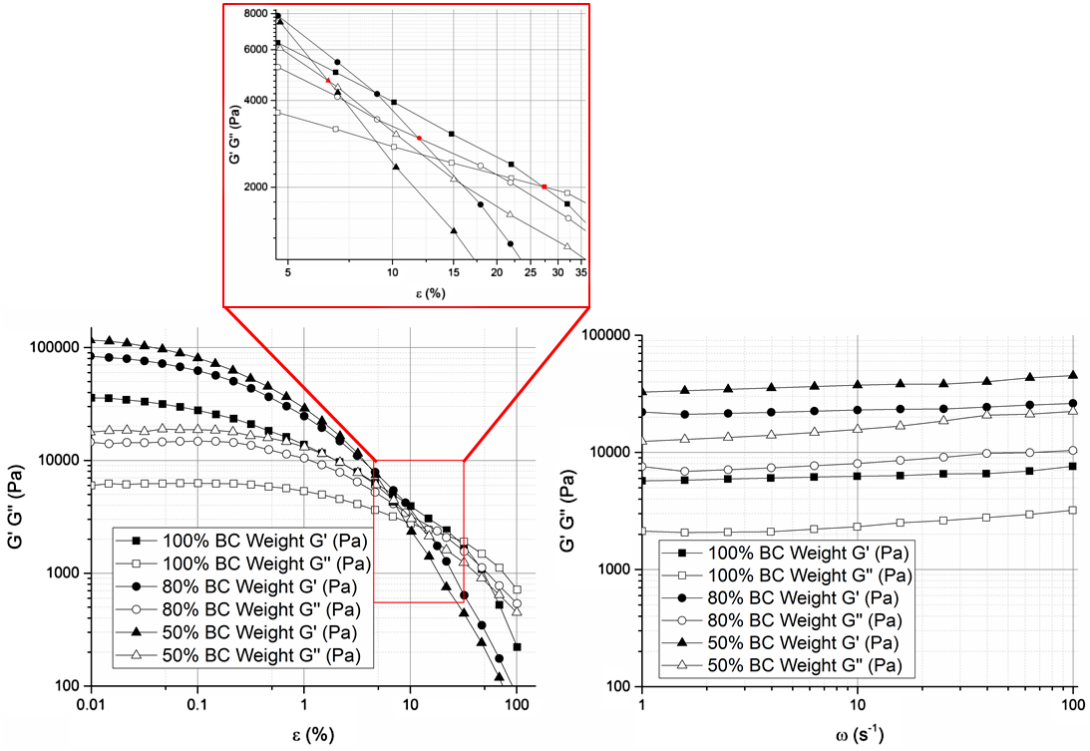
Storage (*G*′) and loss (*G*″) moduli of BC membranes holding 100, 80 and 50% water, measured as a function of strain (left) and frequency (right).

Notes: The red dots indicate the interceptions between *G*′ and *G*″.

Figure 1 displays representative data of the viscoelastic properties of BC membranes (2.3 ± 0.4) mm thick and with different water contents. The viscoelastic properties followed the same pattern for the varied water contents, with higher storage modulus magnitude than that of loss modulus over angular frequency and nearly all the strain range measured. It is also clear that all moduli got higher with lower water contents in BC membranes. Over the applied strain, the storage and loss moduli remained reasonably constant up to 0.1 and 1% strain, respectively – Linear Viscoelastic Region – and then they started decreasing gradually until the loss modulus *G*″ intersected the storage modulus *G*′. With reduction of water content in BC membranes, both moduli were increased and the intersection between *G*′ and *G*″ occurred at lower strain values. Establishing a steady-strain at the limit of the plateau region (1%) led to a slight but almost insignificant increase in the moduli (storage and loss) measured in the frequency range of 1–100 rad/s.

### 3.2. Electrochemical properties

**Figure 2.**
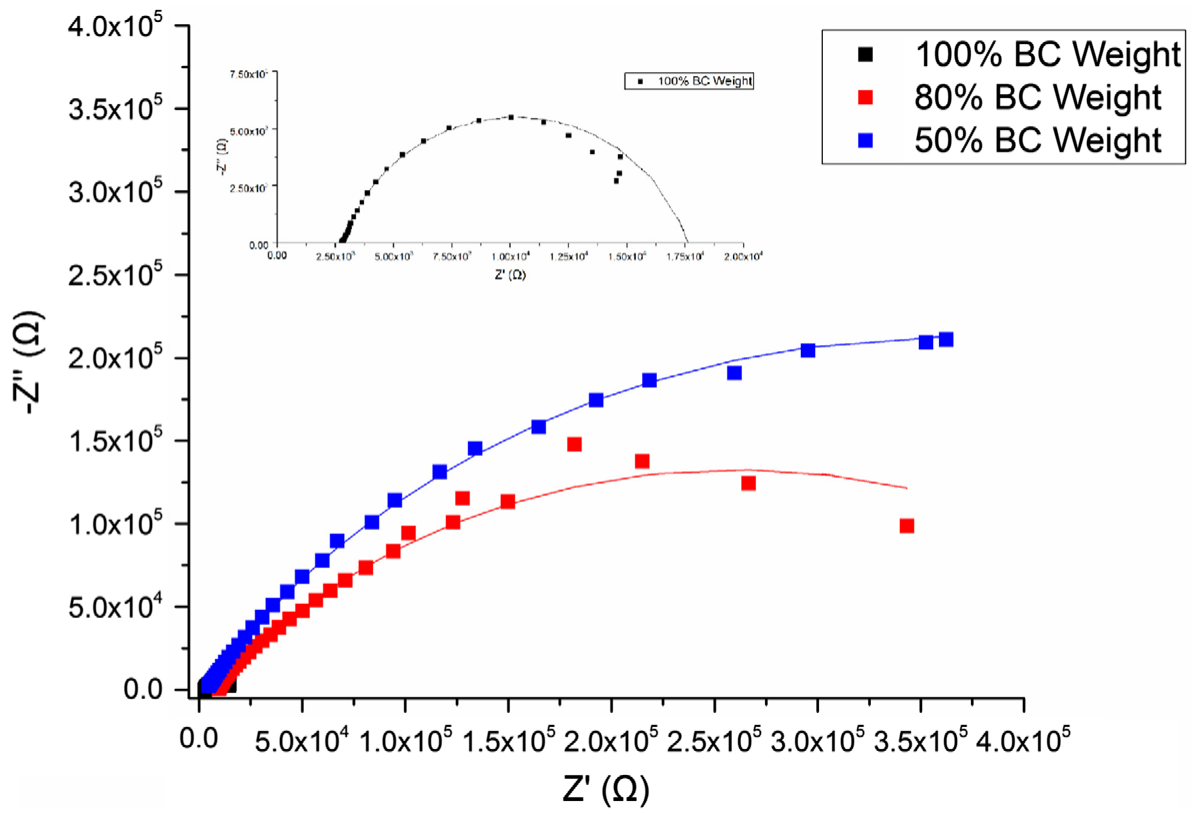
Representative Nyquist plots of BC membranes with 100% (never-dried membrane), 80 and 50% water content.

**Figure 3.**
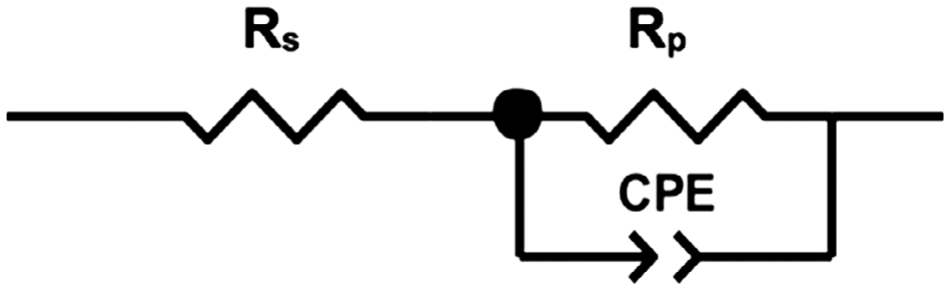
Randles circuit used for fitting the experimental data.

Notes: The solid lines (─) represent the respective fittings.

Figure 2 illustrates the Nyquist plots of BC membranes holding 100, 80 and 50% water content, evincing a semicircle shape that could be fitted into a Randles circuit as shown in Figure 3. This circuit comprises a resistance, Rs (bulk resistance), in series with other resistance, Rp (polarisation or charge transfer resistance), which in turn is in parallel with a constant phase element (CPE). These three parameters can be easily deduced from the Nyquist plots. Rs is obtained from the first intercept of the semicircle with the real axis at higher frequencies and is a measure of the electrolyte resistance. Rp is deduced by the second intercept of the semicircle with the *X* axis (*Z*′) at low frequencies that refers to Rs + Rp, which counts for the resistance of charge transfer between the electrode and the electrolyte. Finally, the CPE quantifies the ability of the material (BC) to store electrical charges (capacitance) and can be inferred from the maximum point of the semicircle. The closer the *N* factor is to the unit (*N* = 1), the closer the element’s behaviour is from that of an ideal capacitor, which is the case under study. Table 1 summarises the corresponding information of the equivalent circuit elements Rs, Rp and CPE.

**Table 1.** Average values of bulk resistance Rs, polarization resistance Rp, constant phase element CPE and *N* values of Randles circuit used to fit the experimental data of six never-dried BC membranes per BC water content (100, 80 and 50%).

BC water content (%)	Rs (kΩ)	Rp (kΩ)	CPE (μF)	*N*
100	3.3 ± 0.6	15.9 ± 0.8	6.2 ± 0.5	≈1
80	5.8 ± 3.0	371 ± 81	1.6 ± 0.6	≈1
50	3.9 ± 1.3	1400 ± 900	1.1 ± 0.8	≈1

By visual inspection of Rs, Rp and CPE values, obvious changes induced by the water content of BC could be observed. The capacitance, CPE, decreased almost six times when BC membranes lost half of the water content, whereas Rp increased about one order of magnitude per 20–30% of water loss from (15.9 ± 0.8) kΩ up to (1400 ± 900) kΩ. In the case of the bulk resistance, Rs, the highest value was measure for 80% of water content (almost 6 kΩ), while for 100 and 50%, lower and identical resistances were observed (below 4 kΩ). Overall, Rp seemed to be the most affected parameter by changes in membrane water content, having risen to around 1000 times when BC lost half of its water, whereas Rs was the least affected and CPE revealed just a slight tendency to change.

### 3.3. Dehydration and numerical modelling of wet BC membranes

**Figure 4.**
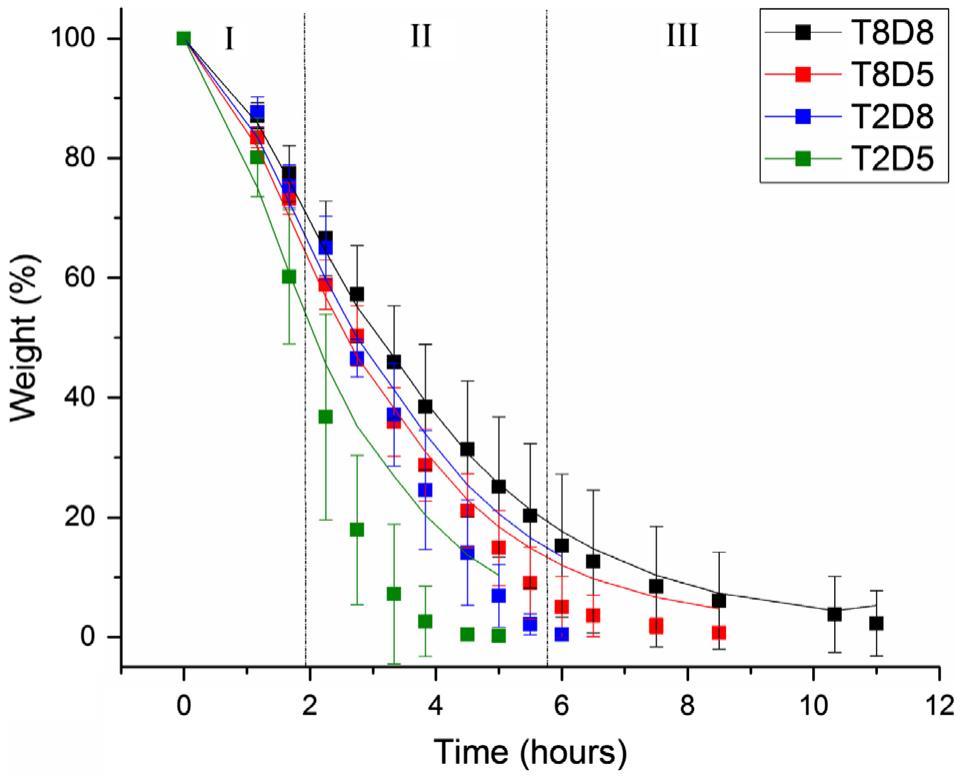
Experimental weight-loss (solid square ■) and respective modelling (solid line ─) of BC membranes 8 mm thick and 8 mm of diameter (T8D8), 8 mm thick and 5 mm of diameter (T8D5), 2 mm thick and 8 mm of diameter (T2D8) and 2 mm thick and 5 mm of diameter (T2D5).

Notes: The weight-loss curves of each size/thickness correspond to an average of five samples and the error bars correspond to the respective standard deviations.

Water evaporation of BC membranes with varied thickness and diameter were analysed after drying in the incubator. The weight-loss profiles over time and their respective predictions by the model equation system 2 are shown in Figure 4 in terms of weight percentage. For all samples, the final weight of the remaining materials after complete water loss was on average 2% of BC initial weight, corresponding to dry cellulose fibres. All profiles displayed the same trend, decreasing over time with distinguishable three phases of water loss in each profile, namely, a slow but short beginning, followed by a fast and sustained weight-loss, and a very slow evaporation rate in the final step. This distribution was mostly observed in the 8 mm thick-samples, which generally required longer time for complete water loss comparing with the 2 mm thick-samples, as expected. Likewise, increasing the diameter from 5 to 8 mm caused slower water evaporation. For instances, the thickest membranes with 8 mm of diameter required roughly 11 h for complete water evaporation, while those with 2 mm of diameter required 9 h.

The curve-fitted weight-loss was obtained using Levenberg–Marquardt algorithm that determined the three parameters values of the system (*k*_1_, *k*_2_ and *k*_3_) that minimised the difference between the predicted and the experimentally observed total mass *M*(*t*) over time (Table 2). The initial proportions for bound and free water were considered based on that only 0.3 of the 98% of water in BC corresponds to free water and the remaining 97.7% is bound water [16]. Thus, at *t* = 0, *B*(0) = 0.977 and *F*(0) = 0.003. In general, the model allows for a very good fitting, with R-squared up to 1.0 (Table 2). The best fitting was observed for the largest membranes, with increased deviation from the model as samples were reduced in size (*R* ^2^ > 0.93). Calculation of the statistical residuals (Figure S2 in supplemented data) corroborated that for T8D8 membranes the theoretical adjustment could be mathematically accepted and for other samples dimensions, the model needed to be finely tuned. Nevertheless, notable conclusions can still be drawn. Table 2 gives the sets of parameter values and respective ratios, calculated by OriginLab software that best fits the experimental data, for each size of membranes.

**Table 2.** Rate coefficients obtained from the model expressed in Equation (2) that gives the best agreement with the experimental data. It is also related to surface area to volume ratio for each sample’s dimension.

	*k*_1_ (hours^−1^)	*k*_2_ (hours^−1^)	*k*_3_ (hours^−1^)	*R*^2^	Surface area/volume (SA/V) (mm^−1^)
T8D8	0.56	11.8 × 10^−8^	0.55	1.0	0.6
T8D5	0.68	1.0 × 10^−8^	0.68	0.99	0.9
T2D8	0.70	65.6 × 10^−8^	0.70	0.95	1.0
T2D5	1.02	20.5 × 10^−8^	1.02	0.93	1.3

From Table 2, it is clear that *k*_1_ and *k*_3_ are much higher than *k*_2_. On average, the un-binding (*k*_1_) and evaporated constant rates (*k*_3_) are about the same regardless the size of the samples, that are meaningfully higher than the binding constant rate (*k*_2_). According to the model, a reduction in the diameter led to an increase of *k*_1_ and *k*_3_ of the thicker membranes in 22% (T8D8 → T8D5) and in 47% (T2D8 → T2D5) of the thinner. In contrast, *k*_2_ decreased in 1000 and 221%, respectively.^1^ Decreasing the thickness of the membranes from 8 to 2 mm led to an increase of the constant rates. In particular, *k*_1_ and *k*_3_ increased by 25% (T8D8 → T2D8) and 50% (T8D5 → T2D5), while *k*_2_ increased in 82 and 95%, respectively.^2^ Because *k*_2_ is very small and it is insignificant as compared to the rate constants *k*_1_ and *k*_3_, variations in *k*_2_ caused by changes in diameter and thickness of the samples, were not considered to be relevant. Overall, the parameters tended to decrease identically with both diameter and thickness, although thickness was apparently the factor that reflected bigger changes. In addition, *k*_1_ and *k*_3_ are proportional to the surface area to volume ratio (SA/V), which means higher SA/V ratios lead to higher un-binding and evaporation rates.

To study further the effect of the geometric parameters on the weight-loss curves, different *k*_1_, *k*_2_ and *k*_3_ from the optimal solution were tested and the weight-loss curves were inspected using the Maple software (Table 3). Increasing *k*_2_ resulted in a more gradual slope, while higher *k*_1_ or *k*_3_ resulted in a steeper slope, and vice versa (Figure 4). Decreasing *k*_2_ to zero did not change the overall trend in the graph preventing that *k*_2_/*k*_1_ and *k*_3_/*k*_1_ are below 1. However, when *k*_2_ was increased by more than one order of magnitude the curve no longer fitted the experimental data for any other combination of *k*_1_ and *k*_3_. Moreover, only small fractional changes in *k*_1_ and *k*_3_ could lead to significant modifications in the fitting.

**Table 3.** Influence of changes to the values of the rate constants in the model.

*k*_1_	*k*_2_	*k*_3_
=	↑	↑
=	↓	↓
↑	=	↓
↓	=	↑
↑	↑	=
↓	↓	=

Notes: = no change, ↑  increase, ↓  decrease.

## 4. Discussion

**Figure 5.**
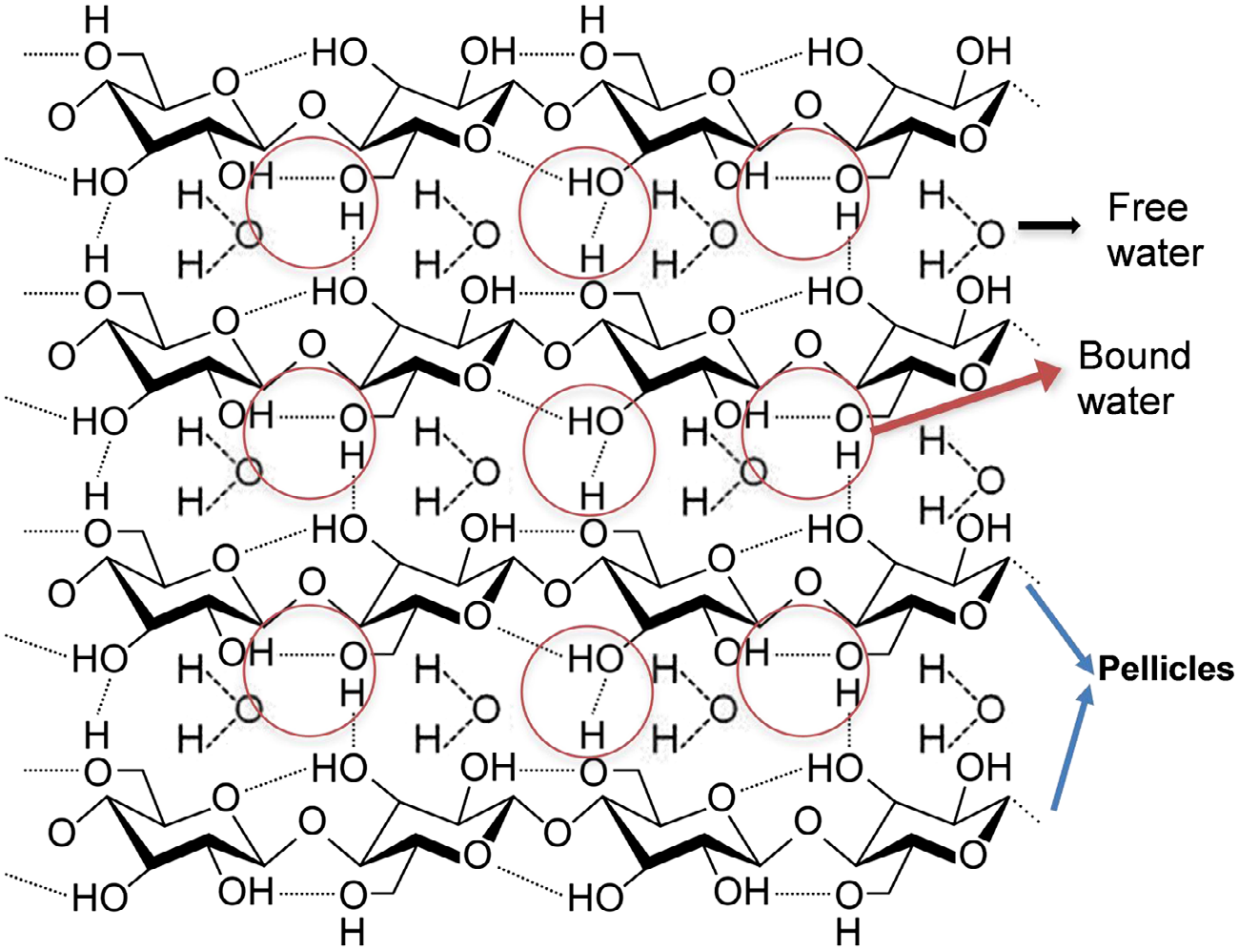
Schematic of the molecular structure of bacterial cellulose and their bound and free water.

BC has a very complex molecular structure that is bounded to water through hydrogen bonding. BC fibrils are formed by linear chains of successive glucan units linked through β-1,4 glycosidic bonds. Inter- and intra-molecular hydrogen bonds are established between glucan chains (See Figure 5), which gives BC a rigid molecular structure but still mechanically flexible [3]. The understanding of the dynamic of the water loss process would allow further engineering of the BC hydration level to tailor various requirements on mechanical and electrochemical properties of BC membrane. Mathematically modelling the water loss can assist the process for reaching such requirements and can guide to a closer perception of the strong correlation between BC network and water. Accordingly, this information may be useful for future manufacturing and optimisation of some biomedical applications such as those for skin (70% water content [17]), cartilage (80% water content [18]) and muscle (75% water content [19]) or for building electrical conductive hydrogels and papers with different conductive range [5].

### 4.1. Effect of water content in the viscoelastic properties

Storage (*G*′) and loss (*G*″) moduli indicate the elastic and viscous responses of BC membranes under compression and shear, allowing for prediction of its mechanical behaviours when used as biomaterials in application such as skin [20], cartilage [18], muscle [21] and blood vessels [22]. Experimentally, the storage modulus quantifies the material’s ability to store energy elastically in entropic distortions of the fibril network of BC membranes; while the loss modulus quantifies the energy that is dissipated.

Depending on the strain and frequency, response of components of BC network, including free and bounded water and BC fibril interaction to shear stress at the interface [23], can be detected via oscillatory rheology. The water content in BC does not dramatically alter the shape of the profiles of both amplitude and frequency sweeps representative of *G*′ and *G*″. The elastic component dominates in the lower strain range, depending on the water content, which leads to the conclusion that BC behaves more like an elastic solid than a viscous liquid at lower strain level. Ideally, a plateau region or Linear viscoelastic region (LVR) should be observed below 1% strain, where the amount of energy (*G*′) stored and damped (*G*″) would be nearly steady and independent of the applied strain.

The LVR of BC could be considered up to 1% strain at which the structure is barely affected and can be fully recovered elastically from the deformation imposed. Thus, 1% strain would be the maximum deformation for reversible structure damage of the fibril network. Stored and lost energies decreased gradually after the plateau region, which could be due to junction disruptions and partially and irreversibly damage to the network. However, BC still responded mechanically more like an elastic material (*G*′ > *G*″). Moreover, the storage and loss moduli got closer with increased strain, which also evinced some loss of the mechanical stability over strain and increased viscous behaviour.

Much of the inherent network properties can be scrutinised during frequency sweep at a given small oscillatory strain close to the plateau region observed in the Amplitude Sweep mode (1% strain) because at this level, membranes store energy elastically between two network points as entropic springs (polymeric segments or strands). This allows a better understanding of the mechanical behaviour of BC with shear deformation. As expected in the LVR, BC fibril in the network structure manifested a solid-like response (*G*′ > *G*″) practically independent on the frequency, as evidenced by that storage and loss moduli reasonably constant throughout the frequency range measured and below the critical strain [24]. The tiny frequency hardening observed could be ascribed to some water loss during testing, although the possibility of frequency dependency could not be totally excluded, in particularly considering the power law dependence of storage modulus *G*′(*w*) ∼ *w* ^0.068^ reported by Clasen C et.al. [14].

When BC was partially dehydrated, fibres aggregated together as result of the evaporation process, leading to some loss of elasticity (or increased rigidity) of more compact and cohesive membranes [14]. This slight mechanical property shift could be witnessed at a first glance in an increase in storage and loss moduli in both sweep modes; but also in a decrease in ‘gel breaking strain’ and in *G*′/*G*″ ratio in the LVR of the frequency sweep (*G*′_100%_/*G*″_100%_ ≈ 2.69; *G*′_80%_/*G*″_80%_ ≈ 2.51; *G*′_50%_/*G*″_50%_ ≈ 2.00). Though for all samples tested BC responded always more like a viscoelastic solid (*G*′/*G*″ > 1), the lesser the water content the closer the properties are to those of a pure solid, and vice versa, which is consistent with the increased stiffness. The storage modulus can be expressed in terms of the density of the polymeric segments:

(4) $$G_{p}^{0}=n/VK_{B}T,$$

where *n* is the absolute number of segments in the total volume *V*, *K*_*B*_ is the Boltzmann constant and *T* is the temperature [14]. In the LVR, with either water loss or swelling, it is anticipated no structural change in the network occurs and the number of segments n should not be affected, but the volume *V* would be altered. Consequently, according to Equation (4), BC network with less water content and lower total volume would have an increase of the storage modulus. Lowering the water content below 50% would lead to a dramatic loss of rheological behaviour with a boost of the storage modulus.

### 4.2. Effect of water content on the electrochemical properties

Electrochemical impedance spectroscopy (EIS) is a very useful tool that allows measurement of electrical properties driven by chemical reactions in non-linear systems, i.e. in systems that do not follow the Ohm’s law (*R* = *V*/*I*). In EIS, the impedance, *Z*, is used to describe the ‘resistance’ of the material instead. Studying the electrochemical properties of BC via EIS might be useful for understanding its contribution to the overall electrical properties of electrical conductive BC and the water content effects. The observed conductive properties extracted from EIS were probably originated from multiple current paths, including cellulose itself, although the protonic conductivity on hydrated surface of BC was most likely the major contributing source, due to the formation of protonic carrier by hydration of the surface of BC nanofibril [25].

The Randles equivalent circuit used to fit the experimental data showed a reasonable fitting. According to its definition (see Figure 3), Rs is related to the bulk resistance of the system, which accounts for the resistance of the solution or electrolyte inside the material, the material between the electrodes and the electrodes [26]. In our case, Rs is the part contributed by the water resistance and the BC network, and should remain the same regardless the water content. The slight fluctuations observed in the average Rs values could be ascribed to the change of the conformation of the BC network with the dehydration. To a lesser extent, the resistance resulted from the contacts of the working electrode and counter-electrode may also account for Rs [5,26]. In our study, we also observed increased Rp with reduced water content in BC, which might be related to the lower amount of proton carriers in the correspondent BC molecular structures, as well as decreased porosity and pore size of BC network after the dehydration ($R\propto 1/{\text{SA}}_{\text{BC fibrils}}$ [27]). Hence, the increased resistivity would be expected with Rp representing an indirect measurement of the network resistance. Considering that CPE was reduced after the dehydration process, it can be concluded that water contributed to the higher capacitance of BC. BC is formed by multiple stratified layers [9] in which each set of two can be seen as a double layer capacitor with water within it as electrolyte. Eventually, a decrease of the protonic carrier and the distance between those stratified layers due to partial water loss might also contribute to reduction of its capacitance (*C* ∝ *Area*).

### 4.3. Understanding water loss process

The mathematical model for analysing the water loss profile within BC at 37 °C matched reasonably well with the whole experimental data. The interdependency of *k*_1_, *k*_2_ and *k*_3_ suggests that it is the balance of the different processes that determines the water loss in the same time frame. The drying process can be considered as a two-step reaction, in which a series of chemical and physical processes take place concurrently. Water evaporation from surfaces, like in an open tank, is a physical process that depends on the temperature, humidity and velocity of the air above the water surface. In our model, this process is easily described by the evaporated constant rate, *k*_3_, which accounts for water that is lost from BC membranes per unit of time. But the un-binding constant rate, *k*_1_, accounts also for the fact that there is a certain amount of water per unit of time that becomes free within the structure, due to the breaking of hydrogen bonds between cellulose and water; while the binding constant rate *k*_2_, accounts for water per unit of time that re-establishes those hydrogen bonds. Therefore, *k*_1_ and *k*_3_ are the constant rates that contribute to speed up water evaporation, whereas *k*_2_ is responsible for slowing it down.

Hence, based on the model herein proposed, the following mechanism underlining water evaporation from BC can be adopted, which involves both the water that is bound to the cellulose molecule via hydrogen bounds, and the water freely circulating within the network (Figure 5). When the temperature is raised up to 37 °C, the constant rates of the system change according to Arrhenius equation ($\text{k}={\text{Ae}}^{-\frac{{\text{E}}_{\text{a}}}{\text{RT}}}$), causing changes in the chemical equilibrium. ‘The first’ water molecules which evaporate are the free ones located on the surface of the membrane. At the same time, as consequence of heat transfer, hydrogen bonds between water and BC start breaking, freeing water molecules. As free water travels within BC towards its surface, the amount of water that evaporates increases. The binding constant rate is very low but may not be zero due to possible air saturation which may lead to re-conversion of yet to be evaporated free water (remaining in the BC) into bound water. Furthermore, it seems SA/V ratio of thicker membranes (0.6 and 0.9 mm^−1^) is inferior compared to that of thinner ones (1.0 and 1.3 mm^−1^), but further study would be required to confirm this. Although a simple model was presented, we believe this model could be further improved to include other factors, influencing the evaporation-drying process of BC membranes, such as the morphological heterogeneity of BC fibril network and its swelling capacity.

## 5. Conclusions

Water comprises about 98% of the BC structure, and slight changes to its content can lead to significant modifications in the overall properties. The water content was shown not only to be responsible for BC’s viscoelastic characteristics, but also for the electrochemical behaviour found. Lower water contents like 80 and 50% caused increased stiffness, while BC resistance to electron transfer became higher and with lower electron capacity. Therefore, BC mechanical and electrochemical properties could be tailored to different biomedical applications by simply varying the water content that may be controlled with the proposed model for the drying process of BC.

## Disclosure statement

No potential conflict of interest was reported by the authors.

## Funding

This work was supported by the FP7 Marie Curie International Research Staff Exchange Scheme (IRSES) project ‘Micro-Multi-Material Manufacture to Enable Multifunctional Miniaturised Devices (M6)’ [grant number PIRSES-GA-2010-269113].

## Supplemental data

Supplemental data for this article can be accessed https://doi.org/10.1080/14686996.2018.1430981.

## Supplementary Material

Supplemented_data.docx

## Footnotes

1 Percentages calculated based on the expression $\frac{k_{D=5mm}-k_{D=8mm}}{k_{D=5mm}}\times 100\%$

2 Percentages calculated based on the expression $\frac{k_{T=2mm}-k_{T=8mm}}{k_{T=8mm}}\times 100\%$
