# Supplementary material for: Dehydration of bacterial cellulose and the water content effects on its viscoelastic and electrochemical properties
Source: Sci Technol Adv Mater. 2018 Mar 9;19(1):203–11. doi: 10.1080/14686996.2018.1430981 (PMC5917443; doi:10.1080/14686996.2018.1430981)
Supplement: Supplemented_data.docx [file TSTA_A_1430981_SM0025.docx]

**Supplemented data**

**Chemical characterisation.** Attenuated total reflection Fourier transform infrared spectroscopy (ATR-FTIR) was performed on BC membranes, which has been dried in an oven at 60 ^o^C for chemical characterisation purposes. The spectrum was acquired in the wavenumber range of 4000-600 cm^-1^, using a Shimadzu FTIR-8400S spectrophotometer (USA) equipped with an attenuated reflectance attachment.

**Chemical characterisation.** Fig. S1 shows the ATR-FTIR spectrum of dried BC membrane. The strong absorption band at 3600-3100 cm^-1^ is assigned to the stretching of -OH groups in BC. O-H bending is also observed at 1641 cm^-1^. CH stretching of CH_2_ and CH_2_ symmetric bending is identified at 2887 cm^-1^ and 1425 cm^-1^, respectively [25]. The peak at 1359 cm^-1^ can be assigned to CC and CO cellulose skeletal vibrations, while that at 1313 cm^-1^ corresponds to stretching and bending modes of hydrocarbons [4]. The absorption peaks at 1161 cm^-1^ and 892 cm^-1^ are ascribed to COC stretching at β-(1-4) linkages. Pronounced peaks at around 1105 cm^-1^ and 1026 cm^-1^ are attributed to vibrational modes of CO stretching groups [25, 26].


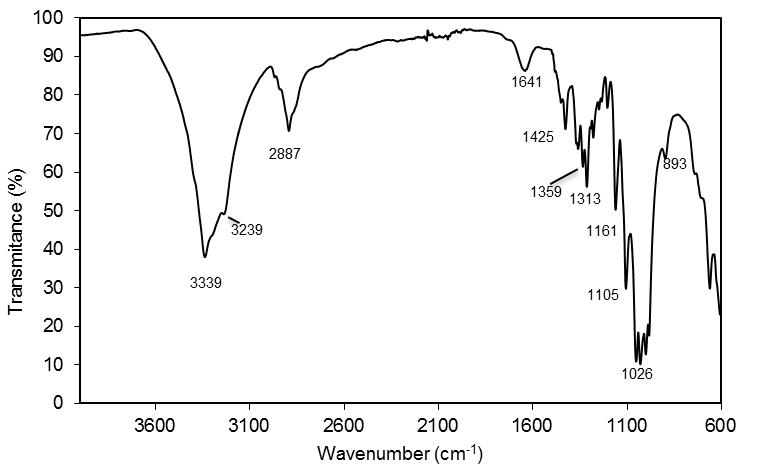


**Figure S1.** ATR-FTIR spectrum of BC.

Figure S2 shows the graphic of the residuals calculated for the weight loss of the samples with different dimensions. The random distribution of the points around zero in T8D8 samples indicates that this is a good model for BC weight loss. However, when the sample size is reduced, the model does not provide so good adjustment since it seems to be a certain degree of autocorrelation

**Figure S2.** Graphics of the residuals of the fitted weight loss (“Y”) for each size of membrane.
